# Ribosomal Protein SA-Positive Neutrophil Elicits Stronger Phagocytosis and Neutrophil Extracellular Trap Formation and Subdues Pro-Inflammatory Cytokine Secretion Against *Streptococcus suis* Serotype 2 Infection

**DOI:** 10.3389/fimmu.2020.585399

**Published:** 2021-02-02

**Authors:** Qiang Sun, Na Li, Li Jia, Wenfei Guo, Hexiang Jiang, Baijun Liu, Chuntong Bao, Mengmeng Liu, Jing Huang, Liancheng Lei

**Affiliations:** ^1^ The Laboratory Department of First Hospital, Jilin University, Changchun, China; ^2^ College of Veterinary Medicine, Jilin University, Changchun, China; ^3^ College of Animal Sciences, Yangtze University, Jingzhou, China

**Keywords:** polymorphonuclear neutrophil, ribosomal protein SA, *Streptococcus suis* serotype 2, blood-brain barrier, infection

## Abstract

*Streptococcus suis* serotype 2 (SS2), an important zoonotic pathogen that causes septicemia, arthritis, and irreversible meningitis in pigs and humans, can be transmitted to humans from pigs. *S. suis* causes huge economic losses to the swine industry and poses a serious threat to public health. Previously, we found that the brain tissues of mice with SS2-induced meningitis showed disrupted structural integrity and significantly enhanced polymorphonuclear neutrophil (PMN) infiltration. We showed that the brain tissues of SS2-infected mice had increased ribosomal protein SA (RPSA)-positive PMN counts. However, the inflammatory responses of RPSA^+^ PMNs to SS2 and their effects on the blood-brain barrier (BBB) remain unclear. Therefore, in studying the pathogenesis of SS2-induced meningitis, it is essential that we explore the functions of RPSA^+^ PMNs and their effects on the BBB. Herein, using flow cytometry and immunofluorescence microscopy analyses, we found that RPSA expression enhances PMN-induced phagocytosis and PMN-induced formation of neutrophil extracellular traps (NETs), which facilitate further elimination of bacteria. PMN surface expression of RPSA also alleviates local inflammation and tissue injuries by inhibiting secretion of the pro-inflammatory cytokines, TNF-α and IL-6. Moreover, the single-cell BBB model showed that RPSA disrupts BBB integrity by downregulating expression of tight junction-associated membrane proteins on PMNs. Taken together, our data suggest that PMN-surface expression of RPSA is a double-edged sword. RPSA+ PMN owns a stronger ability of bacterial cleaning and weakens inflammatory cytokines release which are useful to anti-infection, but does hurt BBB. Partly, RPSA+ PMN may be extremely useful to control the infection as a therapeutic cellular population, following novel insights into the special PMN population.

## Introduction

*Streptococcus suis* infection is a zoonotic disease mainly attributable to *S. suis*, which is distributed worldwide, especially in countries with an extensive swine industry, and is highly prevalent in Africa and Southeast Asia (1). There have been more than 1,500 reported cases of *S. suis* infections worldwide, of which the majority were reported in Thailand, followed by Vietnam. Furthermore, there were major outbreaks of the disease in China in 1998 and in 2005 (2). *S. suis* can be categorized into 35 serotypes based on differences in the capsular polysaccharide (CPS) antigens, among which *S. suis* serotype 2 (SS2) is the most pathogenic and widely distributed serotype (3). SS2 enters the bloodstream in various ways (e.g., *via* the disruption of mucosal tissues), causing symptoms, including meningitis, arthritis, septicemia, and even sudden death in pigs with a mortality rate of more than 10%. As a result, SS2 has caused huge economic losses to the swine industry and has become one of the major infectious diseases affecting the swine industry in China (4). SS2 is a zoonotic pathogen that poses a significant threat to public health as it can be transmitted to humans from pigs *via* direct contact or consumption of undercooked infected pork, leading to diseases, such as bacterial endocarditis, meningitis, and toxic shock syndrome, and may even cause death in severe cases (5). SS2 may cause bacterial meningitis in both pigs and humans, eventually resulting in severe central nervous system (CNS) lesions and death with a mortality rate of 68% (6, 7). However, the mechanism by which the disease progresses to meningitis and its sequelae remain unclear. The effective prevention and treatment methods for *S. suis*-induced meningitis can be determined by exploring its pathogenesis.

Ribosomal protein SA (RPSA) is an important component of the small ribosomal subunit and plays crucial biological roles in cell growth, survival in adverse environments, cell migration, protein synthesis, cell proliferation, and differentiation, among others (8, 9). However, recent studies found that RPSA is involved in the onset of certain diseases. It has been pointed out that RPSA can potentially be used as an alternative therapeutic tool against tumors as it can regulate telomerase activity and it can inhibit telomerase activity by blocking metastasis, promoting angiogenesis, and inducing apoptosis (10, 11). Additionally, RPSA is ubiquitously expressed across various cell types and has been reported as a cell receptor for infectious agents that cause meningitis, including, but not limited to, *Neisseria meningitis*, *Streptococcus pneumoniae*, *Haemophilus influenzae*, and *Escherichia coli*. RPSA expressed on the surface of cerebrovascular endothelial cells may facilitate bacterial invasion *via* ligand-receptor binding to specific bacterial virulence factors (e.g., PilQ and PorA in *N. meningitis*, OmpP2 in *H. influenzae*, CbpA in *S. pneumoniae*, and CNF1 in *E. coli*) (12, 13).Many pathogens infect host cells *via* RPSA, for instance, the prion protein (PrP) that causes spongiform encephalopathy is internalized by binding to RPSA, which has also been reported to interact specifically with dengue virus serotypes (14).

Neutrophils, also known as polymorphonuclear leukocytes (PMNs), are the most abundant white blood cells (WBCs) in the blood. Activated neutrophils are exudated and chemotactically recruited to the infected sites, where they kill pathogens by phagocytosis, degranulation, neutrophils extracellular trap (NET) formation, and cytokine secretion (15). In recent years, increasing evidence has shown that neutrophils are associated with the pathogenesis of various human diseases, including, but not limited to, infectious diseases, sepsis, lung diseases, chronic autoimmune and inflammatory diseases, and cancer (16, 17). In recent years, RPSA has also been reported to be expressed on the surface of CD34^+^ cells in bone marrow and peripheral blood, and promote the homing of these cells to the bone marrow. In addition, RPSA is also expressed on the surface of neutrophils, mononuclear cells, and activated T cells. Interestingly, RPSA can be detected on the surface of neutrophils in the synovial fluid of both healthy individuals and patients with rheumatoid arthritis, but the latter group show decreased expression of RPSA compared to the former, suggesting that its downregulation may favor the onset of rheumatoid arthritis (18, 19). Our preliminary studies have revealed that the brain tissues of SS2-infected mice have significantly enhanced PMN infiltration and significantly upregulated RPSA expression. We speculate that the increased expression of RPSA in brain tissues following SS2 infection may be related to enhanced chemotaxis of PMNs to brain tissues, but the association between RPSA and PMNs in infectious diseases has not yet been reported.

In this study, we constructed a mouse model of SS2 infection and analyzed the changes in RPSA^+^ PMN counts in the brain tissues of SS2-infected mice using histopathological methods and flow cytometry. Furthermore, the bone marrow PMNs of mice were isolated and co-cultured *in vitro* with SS2 to determine changes in RPSA^+^ PMNs over time and analyze its phagocytic capacity, its ability to form NETs and secrete inflammatory cytokines, as well as its damaging effects on the single-cell BBB model. Herein, we provide the necessary foundation for understanding the role of RPSA^+^ PMNs in SS2 infections.

## Materials and Methods

### Mice and Bacterial Strains

C57BL/6 mice were purchased from Liaoning Changsheng Biotechnology Co., Ltd. (permit number: 211002300049079). All animals were euthanized prior to neutrophil isolation. All efforts were made to minimize animal suffering throughout the study. All animal experimental procedures were performed in strict accordance with the Regulations for the Administration of Affairs Concerning Experimental Animals approved by the State Council of the People^’^s Republic of China (1988.11.1).

### Bacterial Strains, and Growth Conditions

The SS2 strain CVCC606 was purchased from the China Veterinary Culture Collection Center and cultured on Tryptic Soy Agar (TSA) plates containing 5% (v/v) fresh fetal bovine serum (FBS). A single colony was transferred into 3 ml of Tryptic Soy Broth (TSB) containing 5% (v/v) fresh FBS and incubated at 37°C for 8 h. The broth culture of SS2 was then four-degree centrifuged for 5 min at 6,000 revolutions and the resulting cell pellet was resuspended in PBS and subject to 10-fold serial dilutions in phosphate-buffered saline (PBS). Diluted bacterial suspensions were subsequently plated on TSA, and the number of colonies were counted to accurately determine the number of SS2 CFUs per milliliter (CFUs/ml).

### Immunohistochemical Analysis

Mouse brain tissues were fixed as previously described (20). Briefly, brain tissues were dehydrated in graded ethanol series, washed in xylene, and embedded in paraffin blocks. Paraffin sections were then embedded, transparentized, and blocked prior to being immunostained overnight at 4°C with the primary anti-RPSA polyclonal antibody dilutions [1:200(v/v)]. Subsequently, the tissue sections were washed and incubated with the streptavidin-peroxidase conjugate at 37°C for 30 min. Tissue sections were then washed and stained with 3,3′-diaminobenzidine (DAB) and hydrogen peroxide (H_2_O_2_). Following thorough rinsing with tap water, the tissue sections were counterstained with hematoxylin, followed by routine dehydration, transparentization, drying, and sealing. Finally, the tissue sections were imaged under a microscope.

### Isolation of PMNs From the Bone Marrow of Mice

The detailed procedures are as follows: 10- to 15-week-old C57BL/6 mice were euthanized *via* cervical dislocation and immersed in 75% (v/v) alcohol for 3–5 min. Next, mice were transferred onto an ultra-clean platform and all subsequent procedures were performed on ice. Briefly, the femur and tibia were retrieved using sterile instruments (i.e., scissors and tweezers) and immersed in D-Hank’s Balanced Salt Solution containing 0.5% (v/v) FBS. After stripping off surrounding muscles and accessory tissues, the medullary cavities of the femur and tibia were rinsed thoroughly and repeatedly with D-Hank’s Balanced Salt Solution (containing 0.5% (v/v) FBS) using a 5 ml syringe until they were completely white. The bone marrow suspension was then thoroughly mixed by repeated pipetting and centrifuged at 1,000 rpm for 5 min. The resulting supernatant was discarded, while the pellet was resuspended in 2 ml of red blood cell (RBC) lysis buffer and added with 8 ml of D-Hank’s Balanced Salt Solution (containing 0.5% (v/v) FBS) to adjust the osmotic pressure. After passing through a 200-mesh screen, the cell suspension was centrifuged in a 15 ml centrifuge tube at 1,000 rpm for 5 min. The resulting supernatant was discarded, while the cell pellet was resuspended in 5 ml of D-Hank’s Balanced Salt Solution and dropped onto 5 ml of 62% (v/v)Percoll PLUS (GE Healthcare, 17544502), followed by centrifugation at 1,000 rpm for 30 min. The centrifugation divided the cell suspension into three layers, among which the upper and middle layers were slowly aspirated and discarded, while 2 ml of the bottom neutrophil layer was slowly aspirated from the bottom of the centrifuge tube and transferred to a fresh 15 ml centrifuge tube. Subsequently, the neutrophil layer was mixed with D-Hank’s Balanced Salt Solution [containing 0.5% (v/v) FBS] and centrifuged at 1,000 rpm for 5 min. After discarding the supernatant, the remaining cell pellet was resuspended in RPMI 1640 medium (Hyclone, USA) for cell counting and cultivation prior to subsequent experiments.

### Construction of C57BL/6 Mouse Model of SS2 Infection

Twenty mice were randomly divided into the following groups; control group, 1 day post-infection group, 2 days post-infection group, and 3 days post-infection group with five mice in each group. C57BL/six mice in the infection groups were infected with 1×10^7^ CFUs of SS2 (grown to the logarithmic phase) *via* intraperitoneal injection, while the control group mice were intraperitoneally injected with an equal volume of physiological saline. Subsequently, brain tissues were harvested at 1, 2, and 3 days post-infection. A total of 0.1 g of brain tissue from each mouse was homogenized with 100 µl of PBS. The brain homogenates were then serially diluted for plate counting and subject to antibody labeling to determine the PMN and RPSA^+^ PMN counts.

### Construction of Single-Cell BBB Model From the hCMEC/D3 Cell Line

The single-cell BBB model was constructed from the hCMEC/D3 cell line using matrices in Transwell inserts (Corning3401). The TEER value remained stable at 130–140Ω×cm^2^ 7 days after seeding, thereby indicating that the model was successfully established (21). ① PMNs in the RPSA-blocking group were blocked with the anti-RPSA antibody (Abcam, ab133645) for 1 h. PMNs in the PMN group and the blank control group were also prepared accordingly. PMNs in each group were then co-incubated with the single-cell BBB model (PMN:hBMEC/D3 = 5:1) for 1 h and 2 h prior to measurement of TEER values, which were recorded for subsequent statistical analyses; ② PMNs in the RPSA-blocking group were infected with SS2 (MOI=2) for 1 h and then treated with 40 µg/ml of gentamicin for another 1 h to eliminate SS2, followed by blocking with the anti-RPSA antibody for 1 h; PMNs in the PMN group were infected with SS2 (MOI=2) for 1 h and then treated with 40 µg/ml of gentamicin for 1 h to eliminate SS2. Additionally, PMNs in the control group were also prepared accordingly. Subsequently, PMNs in each group were co-incubated with the single-cell BBB model (PMN:hBMEC/D3 = 5:1), whose TEER values were measured at hourly intervals up to 6 h post-incubation. Moreover, expression of the tight junction-associated membrane proteins, CDH5 and ITGB1, and the inflammatory permeability factor, CCL2, were determined at 1 h and 2 h post-infection. All of the above data were recorded for subsequent statistical analyses.

### Flow Cytometric Determination of PMN Purity

According to a previously described method (22), the bone marrow PMNs of mice were isolated and resuspended in fluorescent washing buffer (PBS:serum=19:1) for cell counting on a hemocytometer. Bone marrow PMNs were then aliquoted into 1.5 ml centrifuge tubes at ≥ 10^6 cells per tube and centrifuged at 1,500 rpm and 4°C for 10 min. After discarding the supernatant, the remaining cell pellet was resuspended in 300 µl of pre-chilled fluorescent washing buffer and centrifuged again to discard the supernatant. The above step was repeated twice and the cell pellet was finally resuspended in 100 µl of pre-chilled fluorescent washing buffer for antibody labeling. The cell suspension was divided into the following groups; blank control group, Ly6G-labeling group, CD11b-labeling group, and double-labeling group for antibody labeling as per the manufacturer’s instructions. Briefly, the cell suspension in each tube was mixed and incubated in the dark at 4°C for 25 min with 0.3 µl of APC anti-mouse Ly-6G Antibody (Biolegend) and 0.5 µl of FITC anti-mouse CD11b Antibody (Biolegend), respectively. After centrifugation at 1,500 rpm and 4°C for 10 min, the supernatant was discarded, while the remaining cell pellet was resuspended in 300 µl of fluorescent washing buffer and centrifuged again to discard the supernatant. The above step was repeated twice, and the resulting cell pellet was finally resuspended in 300 µl of fluorescent washing buffer. Subsequently, the labeled cell suspensions were transferred into flow cytometry tubes and analyzed using a flow cytometer (BD FACS Calibur, USA).

### Functional Assessment of RPSA^+^ PMNs

Bone marrow PMNs isolated from mice were inoculated into 6-well plates at 10^5 cells per well. After adhering to the wells, the PMNs were infected with SS2 (MOI=2:1) for 30 and 60 min, respectively. Additionally, a control group was also included in this experiment. ① The phagocytic activity of RPSA^+^ PMNs was determined as follows. Pre-treatment with fluorescent microspheres: briefly, the working solution of fluorescent microspheres was prepared by adding 10 µl of fluorescent microsphere stock solution into 10 ml of RPMI 1640 medium containing 1% (v/v)bovine serum albumin (BSA), followed by incubation in the dark at 37°C for 30 min. Next, 1 ml of fluorescent microsphere working solution was added to each of the wells and incubated in the dark at 37°C for 1 h. Adherent cells in each well were washed 3 times with PBS to remove non-phagocytosed free fluorescent microspheres and harvested *via* trypsin digestion in the dark into 1.5 ml centrifuge tubes, which were then centrifuged at 1,000g for 5 min. After discarding the supernatant, the remaining cell pellet in each tube was resuspended in 100 µl of fluorescent washing buffer and incubated in the dark at room temperature for 20 min with 1 µl of anti-RPSA monoclonal antibody (Abcam, ab133645). A blank group was also prepared accordingly for each tube. The labeled cell suspensions were centrifuged at 1,000g for 5 min to discard the supernatant. The above step was repeated twice and the resulting cell pellet was then resuspended in 100 µl of fluorescent washing buffer and incubated in the dark at room temperature for 20 min with 1 µl of Alexa Flour 594-conjugated goat anti-rabbit secondary antibody (Biolegend). Subsequently, labeled cell suspensions were centrifuged at 1,000g for 5 min to discard the supernatant. The above step was repeated twice and the resulting cell pellet was finally resuspended in 200 µl of fluorescent washing buffer. Next, the labeled cell suspensions were transferred into flow cytometry tubes and analyzed using a flow cytometer (BD FACS Calibur, USA). ② After antibody blocking of RPSA, the phagocytic activity of RPSA^+^ PMNs was determined as follows: after adhering to the wells, the bone marrow PMNs isolated from mice were blocked with the anti-RPSA antibody for 1 h. After discarding the supernatant, the adherent cells were exposed to SS2 (MOI=2) for 30 and 60 min, respectively. The subsequent procedures were carried out as described in ①. ③ Secretion of cytokines by RPSA^+^ PMNs: after being counted on a hemocytometer, the PMNs were aliquoted into 1.5 ml centrifuge tubes at ≥ 10^5 cells per tube and centrifuged at 1,500 rpm and 4°C for 10 min. The resulting supernatant was discarded, while the cell pellet was resuspended in 300 µl of pre-chilled fluorescent washing buffer and centrifuged again to discard the supernatant. The above step was repeated twice, and the resulting cell pellet was then resuspended in 100 µl of pre-chilled fluorescent washing buffer and divided into blank control group, IL-6-labeling group, RPSA-labeling group, TNF-α-labeling group, and triple-labeling group for antibody labeling as per the manufacturer’s instructions. Briefly, the cell suspension in each tube was mixed and incubated at room temperature for 25 min with 1.25 µl of PerCP/Cy5.5 anti-mouse TNF-α Antibody (Biolegend), 1.25 µl of PE anti-mouse IL-6 antibody (Biolegend), and 1 µl of RPSA polyclonal antibody. The labeled cell suspensions were then centrifuged twice at 1,500 rpm for 10 min to discard the supernatant, and the resulting cell pellet was resuspended with fluorescent washing buffer and incubated at room temperature for 25 min with 1 µl of goat anti-rabbit IgG/FITC antibody. Next, the labeled cell suspensions were centrifuged twice at 1,500 rpm for 10 min to remove the supernatant. Subsequently, the cell pellet was resuspended in 200 µl of fluorescent washing buffer and transferred into flow cytometry tubes for analysis on a flow cytometer. ④ The formation of NETs by RPSA^+^ PMNs: PMNs were exposed to SS2 (MOI=2) and 100 nM phorbol 12-myristate 13-acetate (PMA, which serves as a positive control) for 2 h. Adherent PMNs in each group were fixed with 4%(v/v) paraformaldehyde for 15 min and washed twice with PBS for 5 min each, followed by blocking at room temperature for 30 min with 10% (v/v) goat serum of the same source as the secondary antibody. After removing the blocking buffer *via* aspiration, the PMNs were incubated overnight at 4°C with the mouse anti-RPSA antibody (1:200), rabbit anti-MPO antibody, and anti-Histone H3 antibody (diluted in 1% (v/v) BSA as per the manufacturer’s instructions). After discarding the diluted antibody solution, the labeled PMNs were washed three times with PBS with Tween-20 (PBST) and incubated for 1 h with diluted fluorescent-labeled secondary antibodies [i.e., FITC-conjugated goat anti-mouse IgG antibody and Per-cy3-conjugated goat anti-rabbit IgG antibody (Biolegend)], in accordance with the manufacturer’s instructions. After being washed 3 times with PBST, the labeled PMNs were stained with the Hoechst nuclear counterstain and mounted with glycerol for immunofluorescence microscopy.

### Measurements of Cytokine mRNA Expression by qPCR

Total RNA was extracted from each sample using the Trizol reagent (Invitrogen) as per the manufacturer’s instructions and subjected to reverse transcription using the Prime Script™ RT Master Mix Kit (TaKaRa) in accordance with instructions provided in the kit. The resulting cDNA was used to determine changes in the transcription levels of target genes. GAPDH was used as the internal reference gene. The results of expression measurement for target genes were normalized using the 2^-△△CT^ method to obtain their relative expression levels. Fluorescence-based qPCR assays were carried out in triplicate for each target gene using the Fast Start Universal SYBR Green Master kit. The polymerase chain reaction (PCR) was conducted using primers (**Table 1**).

**Table 1:** Primer sequences for target genes in this study.

Gene	Amplicon size (bp)	Primer sequences
CDH5 (Human)	86	TTGGAACCAGATGCACATTGAT TCTTGCGACTCACGCTTGAC
ITGB1 (Human)	128	CCTACTTCTGCACGATGTGATG CCTTTGCTACGGTTGGTTACATT
CCL2 (Human)	190	CAGCCAGATGCAATCAATGCC TGGAATCCTGAACCCACTTCT
GAPDH (Human)	101	ACAACTTTGGTATCGTGGAAGG GCCATCACGCCACAGTTTC
IL-6 (Mouse)	134	CTTCCATCCAGTTGCCTTCT CTCCGACTTGTGAAGTGGTATAG
TNF-α (Mouse)	81	ACCACGCTCTTCTGTCTACT GATGAGAGGGAGGCCATTTG
GAPDH (Mouse)	95	AGGTCGGTGTGAACGGATTTG GGCTGTGTAGGAATGGACTG

### Statistical Analyses

The comparison for statistical differences between groups was carried out using one-way analysis of variance (ANOVA). The data are presented as the mean ± standard error of the mean (SEM) of three independent replicates. A *P* value < 0.05 (*) suggests statistical significance; *P* < 0.01 (**) suggests very significant differences; *P* < 0.001 (***) suggests extremely significant differences.

## Results

### RPSA+ PMNs Accumulate in the Brain Tissues of SS2-Infected Mice

**Figure 1:**
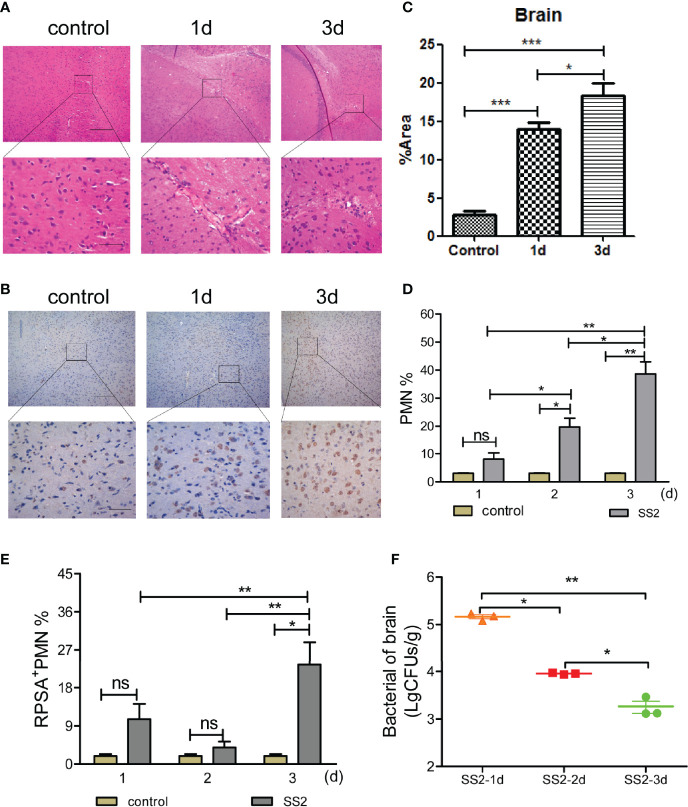
Detection of the polymorphonuclear neutrophil (PMN) and bacterial load in the brain after infected with SS2. **(A)** Pathological changes in brain tissue were observed by HE staining. The scale bar represents 100 μm (top), 400 μm (bottom). **(B, C)** Immunohistochemistry was used to observe the pathological changes in the brain tissues of infected mice. The scale bar represents 100 μm (top), 400 μm (bottom). **(D)** The infiltration of neutrophils in the brain homogenates of mice (n=5) was detected by flow cytometry. **(E)** The expression of ribosomal protein SA (RPSA) on the surface of neutrophils in brain homogenates was detected by flow cytometry. **(F)** Brain tissue bacterial load. Five mice were used in each group for each experiment. *p < 0.05, **p < 0.01, ***p < 0.001 compare with control group, ns, no significant difference.

Bacteria enter the meninges mainly through the blood and penetrate the BBB, causing damages to the CNS (23). In this study, C57BL/6 mice were intraperitoneally injected with 10^8 colony forming units (CFUs) of SS2 (multiplicity of infection, MOI=10) to generate a mouse model of SS2-induced meningitis. Mice were euthanized 24 h and 72 h post-infection to harvest their brain tissues for sectioning, hematoxylin and eosin (HE) staining, and to make observations by microscopy. Brain tissues of infected mice had an extensive infiltration of inflammatory cells (including lymphocytes, monocytes, and neutrophils, of which neutrophils predominated) compared to the non-infection group (**Figure 1A**). Additionally, immunohistochemical analysis on brain tissue sections showed that the brain tissues of infected mice were structurally disrupted compared to the control mice. There was a massive amount of RPSA expressed on the surface of inflammatory cells infiltrating their brain tissues, whose RPSA^+^ areas were several dozen-fold greater than the control group mice and expanded in a time-dependent manner. Moreover, the RPSA^+^ areas were mainly localized on the surface of PMNs (**Figures 1B, C**).

PMNs are one of the important components of the innate immune system. During bacterial infections, PMNs, which serve as the first line of defense, are the first to enter the infected site to kill the invading bacteria. To investigate whether PMNs infiltrate the brain tissues of mice with SS2-induced meningitis, we determined the PMN and RPSA^+^ PMN counts in brain homogenates of mice with SS2-induced meningitis *via* flow cytometry. The results showed that the PMN cell count in the brain tissues of infected mice did not change significantly 1 day post-infection and was significantly greater than that of the control mice 2 and 3 days post-infection. Additionally, the PMN cell count in the brain tissues of infected mice increased in a time-dependent manner (**Figure 1D**), which was consistent with the results of immunohistochemical analysis on their brain tissue sections. The RPSA^+^ PMN cell count in the brain tissue of infected mice did not change 1 and 2 days post-infection, but at 3 days post-infection, their RPSA^+^ PMN cell count increased significantly compared to the past 2 days and was significantly higher compared to the control group (**Figure 1E**). We also found that the bacterial loads in the brain homogenates of infected mice peaked at 1-day post-infection and gradually decreased at 2 and 3 days post-infection (**Figure 1F**). Our data show that the brain tissues of SS2-infected mice display enhanced PMN infiltration with increased RPSA expression on the surface of PMNs, which have a greater ability to eradicate bacteria.

### SS2 Induces RPSA Expression on Bone Marrow PMNs

*In vivo* experiments revealed that the brain tissues of SS2-infected mice had significantly enhanced PMN infiltration and significantly higher RPSA expression on the surface of PMNs. To further validate the correlation between SS2 infection and RPSA expression on the surface of PMNs, the mouse bone marrow neutrophils were isolated with a purity of 94.25% (**Figure S1A**). Additionally, the isolated mice PMNs were subject to nuclear staining using Hoechst, a cell-permeable nucleic acid dye. Fluorescence microscopy revealed that more than 90% of the PMNs had multilobed and kidney-shaped nuclei (**Figure S1B**). Wright-Giemsa staining, which is a commonly used staining method for bone marrow cells, revealed that the bone marrow PMNs isolated from those mice had horseshoe-shaped and kidney-shaped nuclei (**Figure S1C**), confirming the successful isolation of PMNs with a purity that met the requirements for subsequent experiments.

**Figure 2:**
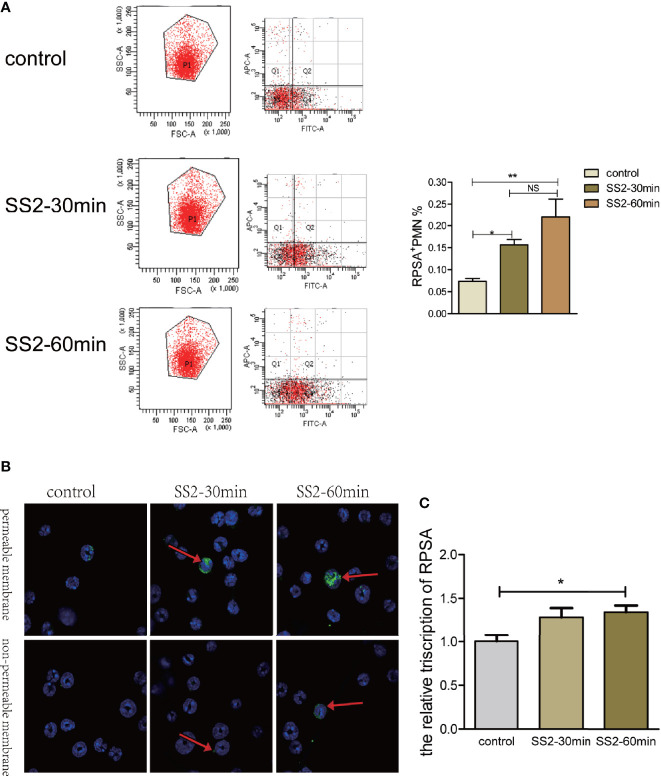
Expression of ribosomal protein SA (RPSA) in polymorphonuclear neutrophil **(**PMN**)** stimulated by SS2. **(A)** RPSA expression on bone marrow-derived neutrophils induced by SS2 was detected by flow cytometry. APC indicates Ly-6G and FITC indicates RPSA. **(B)** RPSA expression on bone marrow-derived neutrophils induced by SS2 was observed by laser confocal microscopy. **(C)** RPSA expression on bone marrow-derived neutrophils was quantified by qPCR. *p < 0.05, **p < 0.01, compare with control group, ns, no significant difference.

RPSA expression on the surface of PMNs was determined *via* flow cytometry following infection with SS2 (MOI=2). The RPSA^+^ PMN cell count of the SS2 infected group was significantly higher than the control group and increased in a time-dependent manner (**Figure 2A**). Since RPSA is widely distributed across cell membrane and nucleus, the distribution of RPSA on neutrophil membrane was observed by laser confocal scanning microscope after immunohistochemical staining. We found that after non-permeable membrane treatment, PMN in SS2 infection group significantly enhanced the membrane localization of RPSA. After transmembrane treatment, the localization of membrane and nucleus of RPSA on PMN was significantly enhanced, showing aggregated distribution (indicated by arrows in **Figure 2B**). Moreover, expression levels of RPSA mRNA in PMNs increased significantly following SS2 infection (**Figure 2C**). *In vitro* assays also confirmed that SS2 can induce expression of RPSA on PMNs (i.e., RPSA^+^ PMNs) which is consistent with our *in vivo* data.

### Expression of RPSA on PMNs Enhances PMN-Induced Phagocytosis and NET Formation

**Figure 3:**
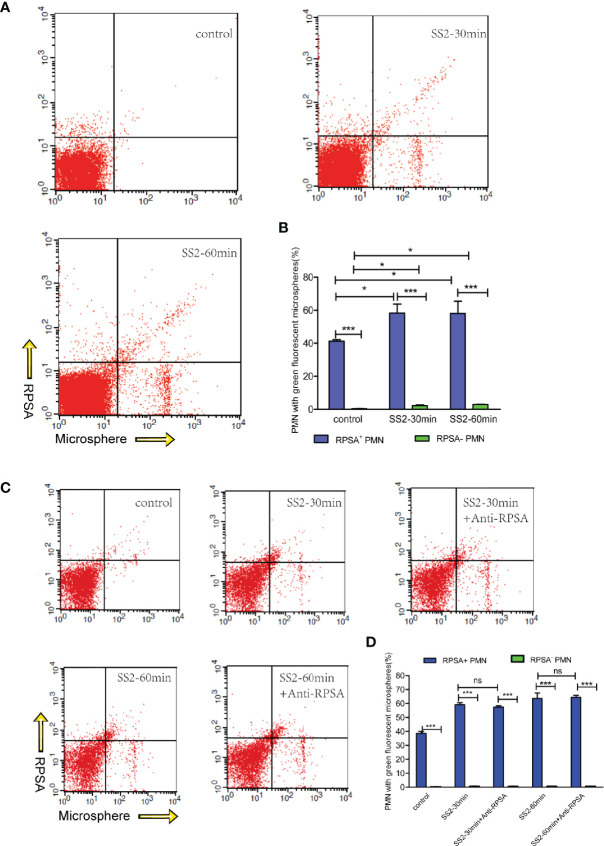
Flow cytometry detection of SS2 phagocytosis by RPSA+ PMN and RPSA^−^ PMN. **(A, B)** Phagocytosis of fluorescent microspheres by RPSA^+^ PMN and RPSA^−^ PMN. X axis represents microspheres and Y axis represents RPSA for bottom panel. **(C, D)** Phagocytosis of fluorescent microspheres by RPSA^+^ PMN and RPSA^−^ PMN after blocking ribosomal protein SA (RPSA). *p < 0.05, ***p < 0.001 compare with control group, ns, no significant difference.

Our *in vivo* animal experimentation and *in vitro* assays revealed that SS2 can induce RPSA expression on PMNs. *In vivo* data also revealed that PMN and RPSA^+^ PMN counts in brain tissues were negatively correlated with bacterial loads. PMNs are one of the most important phagocytic cells in the immune system and kill pathogens mainly *via* phagocytosis and NET formation. Therefore, we speculate that RPSA may be involved in bacterial eradication by regulating the phagocytic and killing capacities of PMNs. The data show that RPSA^+^ PMNs and RPSA^−^ PMNs that have been infected by SS2 had greater phagocytic capacities than the control group (**Figures 3A, B**). Additionally, RPSA^+^ PMNs outperformed RPSA^−^ PMNs in terms of their phagocytic capacity, but there was no difference in their phagocytic capacities following antibody inhibition of RPSA (**Figures 3C, D**), further confirming that SS2 can induce PMNs expression of RPSA, thereby improving phagocytosis. However, the blocking of the RPSA surface epitope did not alter the phagocytic capacity of PMNs. Hence, the specific mechanism by which RPSA regulates the phagocytic capacity of PMNs requires further validation.

**Figure 4:**
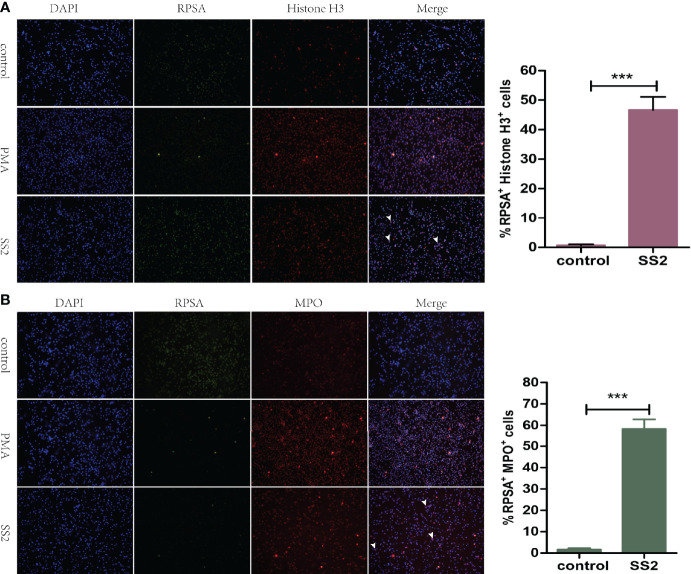
Evaluation of the formation of NET by RPSA^+^ PMN. **(A)** The localization of ribosomal protein SA (RPSA) and Histone H3 was observed using a 20× low power immunofluorescence microscope. **(B)** RPSA and myeloperoxidase (MPO) localization was observed using a 20× low power immunofluorescence microscope. RPSA (green) and MPO/Histone H3 (red) double-immuno-positive cells as indicated by the arrow. ***p < 0.001 compare with control group.

NET is another approach by which neutrophils kill bacteria. Myeloperoxidase (MPO) and Histone H3, as antimicrobial proteins attached to the surface of NET, have strong bactericidal ability (24, 25). Subsequently, we explored the ability of SS2-infected RPSA^+^ PMNs to form NETs. Immunofluorescence microscopy revealed that RPSA and Histone H3 were co-localized on the surface of SS2-infected PMNs with a higher fluorescence intensity than the control group (**Figure 4A**). Moreover, myeloperoxidase and RPSA were also co-localized on the surface of SS2-infected PMNs with a significantly enhanced fluorescence intensity (**Figure 4B**). Therefore, the expression of RPSA on the surface of SS2-infected PMNs enhanced its ability to secrete NETs (i.e., bactericidal proteins MPO and Histone H3), further showing that RPSA can enhance the phagocytic and killing capacities of PMNs.

### SS2 Infection Inhibits the Secretion of Pro-Inflammatory Cytokines by RPSA+ PMNs

**Figure 5:**
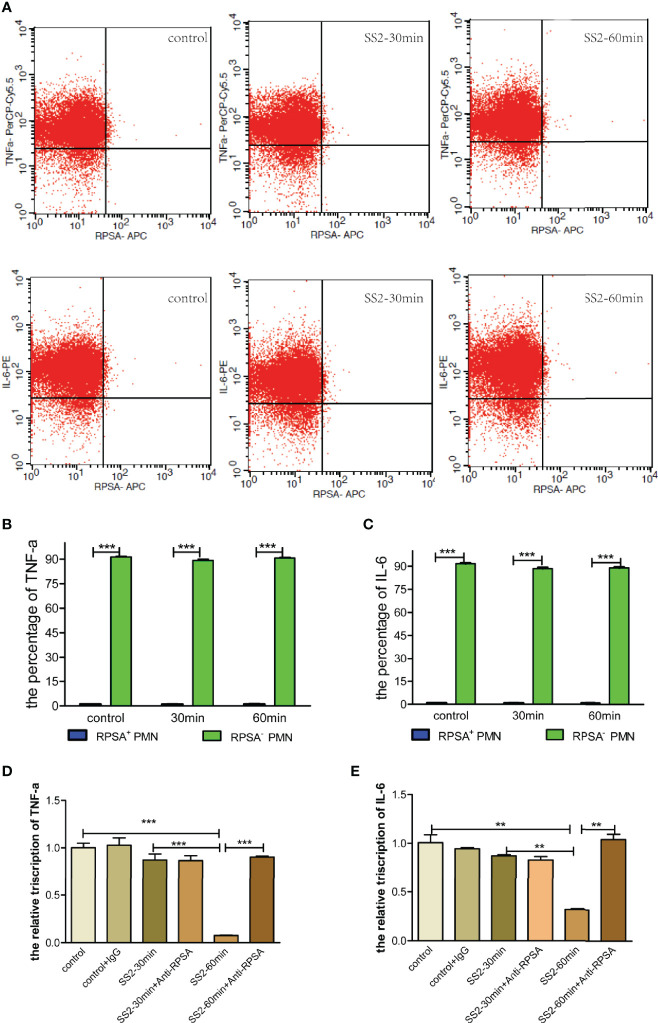
Comparison of TNF-α and IL-6 secreted by RPSA^+^ PMN and RPSA^−^ PMN. **(A–C)** Secretion of TNF-α and IL-6 by RPSA^+^ PMN and RPSA^−^ PMN were detected by flow cytometry. **(D, E)** Secretion of TNF-α and IL-6 by RPSA^+^ PMN and RPSA^−^ PMN were detected by qPCR. **p < 0.01, ***p < 0.001 compare with control group.

SS2-infected PMNs were subject to flow cytometry to investigate cytokine secretion by RPSA^+^ PMNs. The results showed that RPSA^+^ PMNs had significantly lower secretion of pro-inflammatory cytokines (i.e., TNF-α and IL-6) than RPSA^−^ PMNs, but there were no differences in the secretion of TNF-α and IL-6 by RPSA^+^ PMNs and RPSA^−^ PMNs between the SS2 infection group and the non-infection group (**Figures 5A–C**). Additionally, the expression levels of TNF-α and IL-6 mRNA increased significantly in SS2-infected PMNs after blocking the RPSA epitope (**Figures 5D, E**). Our data demonstrate that the SS2-induced expression of RPSA on PMNs can alleviate local inflammatory injuries by inhibiting TNF-α and IL-6 secretion in PMNs.

### RPSA+ PMNs Disrupt BBB Permeability

Histopathologicaly of brain tissue sections from SS2-infected mice in our preliminary animal studies revealed cerebral hyperemia and edema with structural disorders, enhanced PMN infiltration and exudation, as well as structural disruption and increased bacterial load in the brain tissues (**Figure 1**). We speculate that RPSA^+^ PMNs may be involved in BBB disruption allowing facilitation of bacterial entry into the brain tissues. Therefore, we constructed an *in vitro* single-cell BBB model using the immortalized human cerebral microvascular endothelial (hCMEC/D3) cell line to investigate the effects of PMNs on the permeability of the BBB and the expression of tight junction-associated membrane proteins. The data show that the PMN-exposed single-cell BBB model had significantly reduced transendothelial electrical resistance (TEER) values and elevated permeability 1 h and 2 h post-exposure. There were no changes in both the permeability of the BBB (**Figure S2A**) or the expression of tight junction-associated membrane proteins (i.e., ITGB1 and CDH5) following antibody-mediated blockade of RPSA (**Figure S2B**, **C**). On the other hand, expression of monocyte chemoattractant protein [chemokine (C-C motif) ligand 2, CCL2] was significantly upregulated 2 h post-exposure and was downregulated following antibody-mediated blockade of RPSA (**Figure S2D**). The above data suggest that PMNs contribute to the disruption of BBB integrity, which can be alleviated by blocking RPSA. This probably occurs *via* inhibition of RPSA-mediated, PMN-induced expression of CCL2, which reduces monocyte chemotaxis and the disruption of brain microvascular endothelial cells (BMEC).

**Figure 6:**
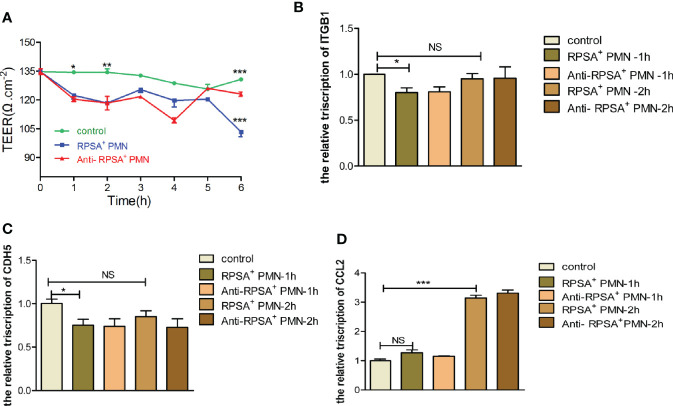
Damage of BBB model by RPSA+PMN induced by SS2. **(A)** Effect of RPSA^+^ PMN on the permeability of the hCMEC/D3 single cell model. **(B–D)** qPCR was used to detect the expression of tight junction proteins in the hCMEC/D3 single cell model by RPSA^+^ PMN. *p < 0.05, **p < 0.01, ***p < 0.001 compare with control group, ns, no significant difference.

Next, we explore the effects of SS2-induced expression of RPSA in PMNs within the BBB, the permeability of the BBB and the expression of tight junction-associated membrane proteins were determined following exposure to PMNs in the RPSA^+^ PMN group, the RPSA-blocking group, and the blank control group, respectively. The results showed that the RPSA^+^ PMN-exposed BBB model had a significantly lower TEER value than the control group 2 h and 6 h post-exposure. In contrast, the TEER value of the BBB model increased significantly following exposure to PMNs in the RPSA-blocking group for 6 h (**Figure 6A**). Additionally, expression of the tight junction-associated membrane proteins, ITGB1 and CDH5, in the RPSA^+^ PMN-exposed BBB model was significantly downregulated 1 h post-exposure (**Figures 6B, C**), while CCL2 expression was significantly upregulated 2 h post-exposure (**Figure 6D**). There were no differences in the expression of CDH5, ITGB1, and CCL2 following RPSA blockade.

In summary, RPSA exacerbates the disruption of BBB integrity by PMNs by downregulating expression of tight junction-associated membrane proteins.

## Discussion

The development of SS2-induced meningitis is mainly triggered by increased inflammatory responses in the CNS following bacterial invasion of the brain tissues. Additionally, meningitis is the result of excessive local inflammation characterized by neutrophil infiltration. Despite being the main innate immune cells implicated in the fight against bacterial infection, the defense mechanism(s) of neutrophils against SS2-induced meningitis remain unclear. In the present study, we have shown the presence of RPSA^+^ PMNs in the brain tissues of mice with SS2-induced meningitis, and demonstrated that RPSA expression regulates PMN function and enhances its phagocytic capacity and ability to form NETs, thereby enabling the PMN-mediated phagocytosis and killing of pathogens. Moreover, RPSA can alleviate local inflammatory injuries by reducing neutrophil-mediated secretion of pro-inflammatory cytokines, such as TNF-α and IL-6. These indicated that RPSA+ PMN is going to be extremely useful to control the infection as a therapeutic cellular population without excessive inflammation. But on the other hands, we also confirmed that RPSA+ PMN can exacerbate disruption of BBB integrity *via* downregulation of tight junction-associated membrane protein expression. Thus, our study has also revealed novel insights into the pathogenesis of bacterial meningitis and provided new theoretical knowledge on the mechanism of BBB disruption.

Ribosomal proteins, the main components of ribosomes in host cells, play an important role in protein biosynthesis. In recent years, there has been an increasing number of studies suggesting that changes in ribosomal biosynthesis may severely affect tissue and cell functions, subsequently causing different diseases (26). The 40S RPSA, is a ribosomal protein that is ubiquitously expressed across almost all cell types with relatively extensive localization in cell membranes, the rough endoplasmic reticulum, the nuclear membrane, among other cellular organelles. RPSA is involved in ribosomal biosynthesis, cytoskeletal organization, and nuclear functions, and plays crucial biological roles in cell growth, survival in adverse environments, cell migration, protein synthesis, as well as cell proliferation and differentiation (27–29). Under abnormal circumstances, RPSA may cause tumor metastasis, neurodegenerative diseases, and abnormal development (30, 31). RPSA distributed on the surface of cellular membranes also mediates infections by pathogens, such as *S. pneumoniae*, *N. meningitis*, *H. influenzae*, *E. coli* K1, and *S. suis*. As a receptor for virulence factors of these bacteria, RPSA enables bacteria to infiltrate the BBB by facilitating entry into cells or by eliciting disruption of the BBB by inducing apoptosis, thereby allowing invasion of brain tissues and, subsequently, bacterial meningitis (32, 33). However, the distribution and the role of RPSA in PMNs, as well as its correlation with infections have not yet been reported. For the first time, we found that as a surface receptor on PMNs, RPSA regulates bacterial load by enhancing the phagocytic and bactericidal activities of PMNs and by reducing secretion of inflammatory cytokines. Nonetheless, the detailed mechanisms involved in these RPSA-mediated effects still require further study. Additionally, RPSA can exacerbate the disruption of BBB integrity by PMNs by downregulating expression of tight junction-associated membrane proteins in the BBB.

Neutrophils are associated with the pathogenesis of various respiratory diseases, such as asthma, chronic obstructive pulmonary disease (COPD), cystic fibrosis, and lung cancer. Extensive neutrophil infiltration and activation will eventually lead to apoptosis and tissue injuries (34). However, the involvement of neutrophils in these processes varies across different diseases and is affected by various factors. Neutrophil activity needs to be appropriately increased if it does not function properly during severe bacterial or fungal infections. On the contrary, some inflammatory diseases are primarily characterized by excessive neutrophil infiltration. Inflammation caused by inappropriate neutrophil activation will result in serious damage to the host tissues. Consequently, functional conversion of neutrophils is regulated by numerous mechanisms (35–37). In recent years, there have been an increasing number of studies showing that neutrophil surface receptors play specific roles in the pathogenesis of some diseases and can affect its functions *via* various mechanisms. For instance, formyl peptide receptors (FPRs) were initially found to be primarily associated with the neutrophil chemotaxis. FPRs, G protein-coupled receptors (GPCRs) found on the surface of PMNs, play a role in infectious and inflammatory diseases. Furthermore, FPRs can regulate neutrophil function *via* different pathways (38). During *Staphylococcus aureus* infections, activation of neutrophils alters surface IL-20R chains, thereby reducing their migratory, phagocytic, and degranulation capacities (39). We found that the phagocytic and bactericidal capacities of RPSA^+^ PMNs were upregulated during SS2 infections. RPSA^+^ PMNs may participate in the disruption of BBB by downregulating the expression of tight junction-associated membrane proteins. These data represent new knowledge which has improved our understanding of PMN surface molecules.

The expression of T-cell receptor (TCR)-based variable immunoreceptors (which can enhance neutrophil-mediated phagocytosis) which are generated *via* V(D)J recombination in neutrophils in the cerebrospinal fluid can be rapidly induced during early stages of bacterial meningitis (40). Moreover, neutrophil surface expression of TGF-β receptor II has been shown to facilitate recruitment of neutrophils to infected sites to promote bacterial elimination in an experimental model of *S. pneumoniae*-induced meningitis (41). Furthermore, during *N. meningitis* infections, the PMN surface expressed calcium-binding protein receptor has been shown to allow *N. meningitis* to evade PMN-induced phagocytosis and death *via* depletion of zinc ions (42). In addition, NETs, which are another major bactericidal mechanism of neutrophils, are mainly comprised of extracellular concentrated DNA and granular proteins (such as MPO and histone) and play an indispensable role in neutrophil function (43, 44). Neutrophils form NETs to kill bacteria during bacterial meningitis. However, bacteria with surface-bound nucleases, such as *N. meningitis* and *Streptococcus* spp., can evade the bactericidal activity of NETs mainly *via* nuclease digestion of DNA (45, 46). Pathogenic *Acinetobacter baumannii* inhibits the formation of NETs by suppressing neutrophil adhesion (47). Our study results showed that as well as enhancing their phagocytosis and bactericidal activities, RPSA expression on the surface of PMNs can also promote formation of NETs. The mechanism by which this occurs may be associated with increased adhesion between bacteria and PMNs, which facilitates NET formation and further elimination of bacteria.

Cytokine secretion is essential for normal neutrophil function during pathogenic infections. Studies have shown that the release of products, such as toxins, peptidoglycans, and other cell wall components, from pathogens that have disrupted the BBB and invaded the CNS, directly or indirectly cause structural damage to the CNS. Moreover, these bacterial products may activate the immune responses and promote infiltration of neutrophils, which generate inflammatory factors (e.g., TNF-α, IL-6, and IL-1) and large amounts of reactive oxygen species (ROS) in the cerebrospinal fluid, resulting in damage to the brain tissues (48, 49). Therefore, the strict regulation of neutrophil-mediated immune responses protect the host against pathogen-induced damage, and they can also prevent harmful inflammation and tissue injuries. In this study, we found that RPSA^−^PMN co-expressed TNF-α and IL-6, while RPSA^+^PMN co-expressed less. Therefore, neutrophils were versatile. Hence, RPSA can alleviate inflammatory injuries by inhibiting the PMN-mediated secretion of the proinflammatory cytokines, TNF-α and IL-6. However, the detailed mechanism(s) by which RPSA regulates secretion of TNF-α and IL-6 by neutrophils requires further in-depth study.

*S. suis* infections may trigger intense neutrophil-dominated responses in the host against invading pathogens in the subarachnoid space (50). A previous study on the mechanism of microglial activation following nervous system injuries found that upregulation of RPSA expression on the surface of microglia following brain injuries may activate microglia and promote its migration to the brain tissues, thereby causing damage to brain tissues (51). RPSA has been reported to mediate disruption of the BBB by various meningitis-inducing bacteria. Clinical symptoms of bacterial meningitis are mainly associated with the increased inflammatory responses. The BBB can be disrupted in different ways, and the host-bacteria interaction may facilitate bacterial invasion of the brain. Fimbrial adhesins enable *S. pneumoniae* to invade the brain by binding to immunoglobulin receptors and platelet endothelial cell adhesion molecules aggregated on the surface of cerebrovascular endothelial cells (52–54). Additionally, BBB integrity can be disrupted *via* secretion of inflammatory cytokines and changes in brain endothelial permeability. Group B *Streptococcus* promotes the production of nitric oxide (NO) and IL-8 by upregulating the expression of inducible nitric oxide synthase (iNOS) (55, 56). *H. influenzae* disrupts the expression of tight junction-associated membrane proteins (57, 58). *Listeria monocytogenes* also can induce BBB disruption by activating the NF-κB signaling pathway in cerebrovascular endothelial cells and disrupting the expression of intercellular adhesion molecule 1 (ICAM-1) and vascular cell adhesion molecule (VCAM-1) (59, 60). SS2 induces cerebrovascular endothelial cells to produce a large amount of inflammatory cytokines, and neutrophils are chemotactically recruited by chemokines to the brain tissues during SS2-induced meningitis. However, excessive neutrophil infiltration may also disrupt BBB integrity (61, 62). In this study, we confirm that PMNs promote destruction of BBB integrity by altering the permeability of cerebrovascular endothelial cells and disrupting the expression of tight junction-associated membrane proteins. Additionally, RPSA expression on the surface of PMNs may exacerbate BBB destruction. Hence, as a surface receptor of PMNs, RPSA can mediate this BBB disruption. These findings have furthered our understanding of the pathogenesis of bacterial meningitis and provided new theoretical knowledge on the mechanism of BBB disruption.

It has been long believed that the immune system is the body’s defense responses, but some scientists have also proposed the “danger” hypothesis, which postulates that the immune system responds to pathogens as well as danger signals released by damaged tissues even in the absence of invading bacteria (63). To understand the interaction between the CNS and the immune system at homeostasis and under disease states, scientists have begun to study the approaches by which immune cells “patrol” the meninges under healthy conditions and invade the brain parenchyma under pathological conditions; the effects of these immune cells on the functions of the CNS; the roles of immune cells following CNS injuries; and the evolutionary correlation between the two systems (64, 65). Therefore, our study has exposed RPSA as a receptor protein that serves as a bridge between the immune system and the CNS, thereby further enriching knowledge on the pathogenesis of SS2 infections.

In summary, we have confirmed that chemotactic recruitment of PMNs to SS2-infected brain tissues may induce RPSA expression. Our *in vitro* studies have also demonstrated that SS2 may induce PMN surface expression of RPSA. We also show that RPSA expression enables elimination of SS2 by increased PMN-induced phagocytosis and NET formation. Moreover, reduced secretion of pro-inflammatory factors (i.e., IL-6 and TNF-α) in RPSA^+^ PMNs can alleviate inflammatory injuries at the infected sites. Furthermore, RPSA^+^ PMNs exacerbated the loss of BBB integrity, which was alleviated following RPSA blockade. Therefore, RPSA^+^ PMN is a double-edged sword. The induced immune responses against SS2 infections require further investigation to reveal further mechanistic insights into disease pathogenesis and potential treatments of SS2-induced meningitis.

## Data Availability Statement

The original contributions presented in the study are included in the article/**Supplementary Material**. Further inquiries can be directed to the corresponding authors.

## Ethics Statement

The animal study was reviewed and approved by the Animal Experimental Ethics Committee of Veterinary College of Jilin University.

## Author Contributions

Designed the experiments: LL and JH. Performed the experiments: QS, NL, BL, LJ, WG, CB, and ML. Conducted the analysis: HJ and CB. Wrote and revised the manuscript: QS, NL, JH, and LL. All authors contributed to the article and approved the submitted version.

## Funding

This study was supported by the National Key R&D Program of China (2017YFD0500204).

## Conflict of Interest

The authors declare that the research was conducted in the absence of any commercial or financial relationships that could be construed as a potential conflict of interest.

## Supplementary Material

Supplementary Figure 1 Isolation and identification of neutrophils from mouse bone marrow. **(A)** The purity of mouse bone marrow neutrophils was identified by flow cytometry. **(B)** The nuclear morphology of mouse bone marrow neutrophils was observed using a 20× low power fluorescence microscope. **(C)** Giemsa staining was used to observe the nuclear morphology of mouse bone marrow neutrophils.

Supplementary Figure 2 Effect of RPSA on PMN on BBB. **(A)** Effect of PMN on the permeability of the hCMEC/D3 single cell model. **(B-D)** qPCR was used to detect the expression of tight junction proteins in the hCMEC/D3 single cell model by PMN.
