# Supplementary figures and images for: Ribosomal Protein SA-Positive Neutrophil Elicits Stronger Phagocytosis and Neutrophil Extracellular Trap Formation and Subdues Pro-Inflammatory Cytokine Secretion Against Streptococcus suis Serotype 2 Infection
Source: Front Immunol. 2021 Feb 2;11:585399. doi: 10.3389/fimmu.2020.585399 (PMC7884477; doi:10.3389/fimmu.2020.585399)

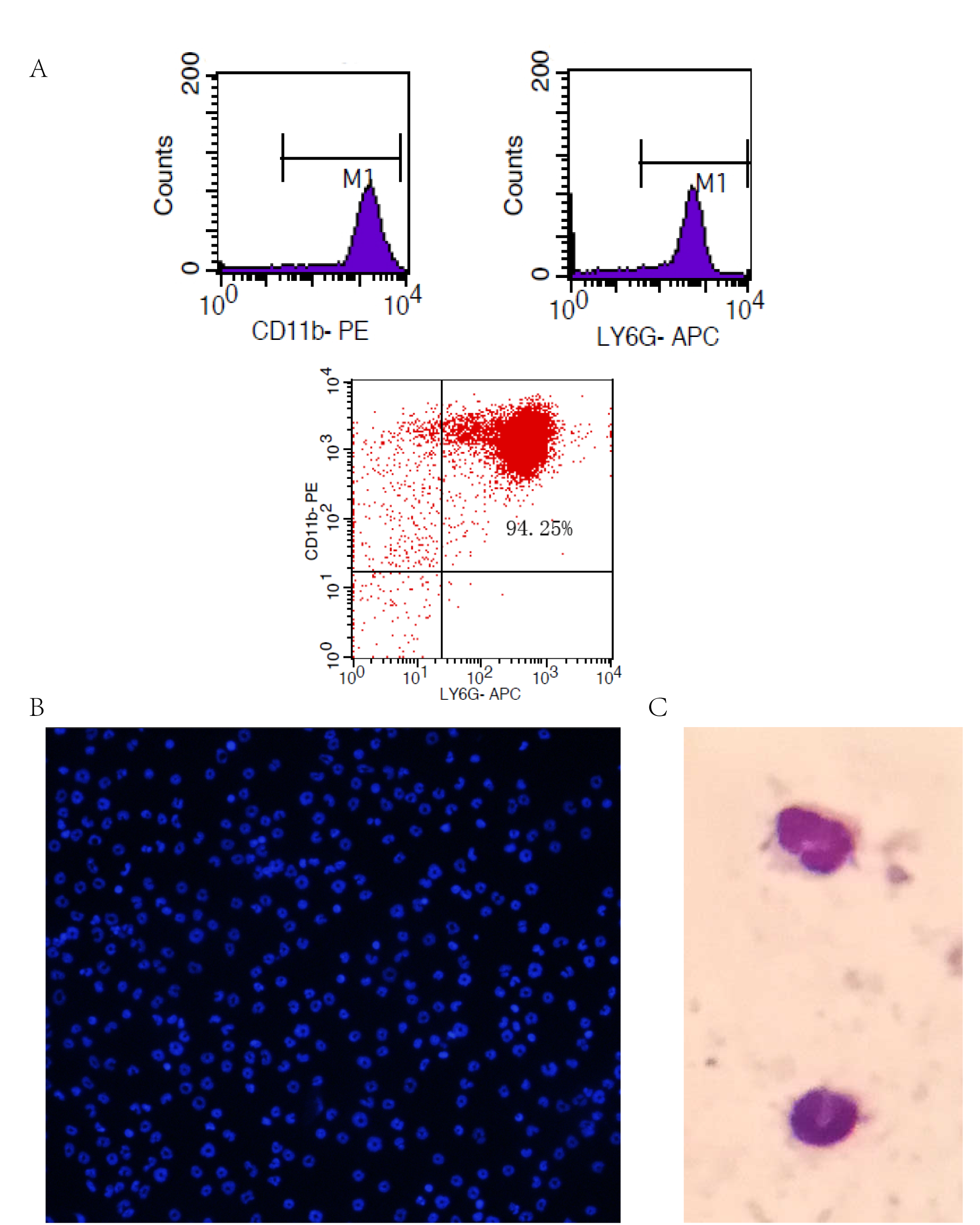

Supplement: Supplementary Figure 1 — Isolation and identification of neutrophils from mouse bone marrow. (A) The purity of mouse bone marrow neutrophils was identified by flow cytometry. (B) The nuclear morphology of mouse bone marrow neutrophils was observed using a 20× low power fluorescence microscope. (C) Giemsa staining was used to observe the nuclear morphology of mouse bone marrow neutrophils. [file Image_1.tif]

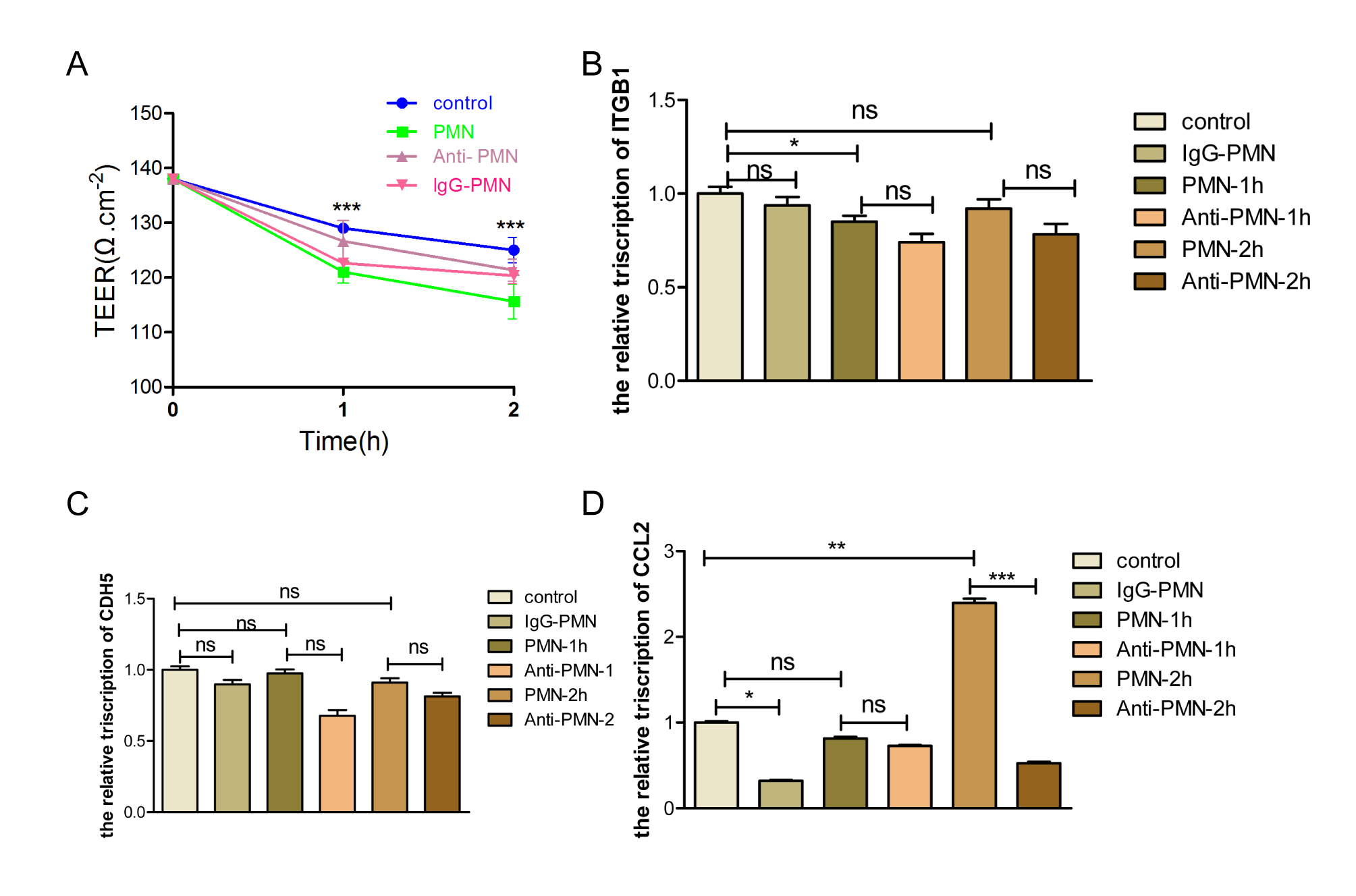

Supplement: Supplementary Figure 2 — Effect of RPSA on PMN on BBB. (A) Effect of PMN on the permeability of the hCMEC/D3 single cell model. (B-D) qPCR was used to detect the expression of tight junction proteins in the hCMEC/D3 single cell model by PMN. [file Image_2.tif]
